# Depressive and anxiety symptom trajectories in psychiatric inpatients with nosocomial infection: a retrospective study from China

**DOI:** 10.3389/fpsyt.2026.1947507

**Published:** 2026-09-24

**Authors:** Liming Huo, Jiaquan Liang, Kexin Tan, Liling Zheng, Hualin Wu, Fangcheng Fan, Caixia Xu

**Affiliations:** 1 Department of Infection Control, The Third People’s Hospital of Foshan, Guangdong, China; 2 Department of Psychiatry, The Third People’s Hospital of Foshan, Guangdong, China

**Keywords:** companion caregiver, hamilton anxiety rating scale, hamilton depression rating scale, nosocomial infection, psychiatric inpatients

## Abstract

**Background:**

Psychiatric inpatients are particularly vulnerable to nosocomial infections (NIs) due to cognitive impairment, impaired self-care, and closed ward environments. This study aimed to characterize the epidemiological and pathogen profile of NI, examine the trajectory of depressive and anxiety symptoms during hospitalization, identify risk factors for non-respiratory infections, evaluate the role of companion caregivers, and quantify the economic burden attributable to NI in a psychiatric hospital setting.

**Methods:**

A retrospective analysis was conducted on 79 NI cases from a psychiatric hospital in China (March 2022 to June 2025). Data included demographics, infection characteristics, hospitalization cost, and paired Hamilton Depression Rating Scale (HAMD) and Hamilton Anxiety Rating Scale (HAMA) scores at admission and before discharge. Statistical analyses included chi-square tests, t-tests, Wilcoxon signed-rank tests, Mann-Whitney U tests, and multiple linear regression.

**Results:**

Respiratory tract infections predominated (60/79, 75.95%). Females had significantly higher non-respiratory infection rates than males (16/44, 36.36% vs 3/35, 8.57%, chi-square=6.792, P = 0.009), and companion-accompanied patients had higher non-respiratory infection rates than those without companions (14/40, 35.00% vs 5/39, 12.82%, chi-square=4.173, P = 0.041). Paired analysis revealed significant improvement in both depressive (HAMD change: −7.00 ± 10.60, paired t=2.723, P = 0.015; Wilcoxon P = 0.015; Cohen’s d=0.66) and anxiety symptoms (HAMA change: −4.19 ± 6.32, paired t=2.652, P = 0.018; Wilcoxon P = 0.029; Cohen’s d=0.66) from admission to discharge, though residual symptoms persisted. Each additional day of NI duration was associated with an additional 730.79 CNY in total hospitalization cost. Multiple regression for cost prediction yielded R²=0.774. Mycoplasma pneumoniae was the most frequently identified pathogen (14/79, 17.72%), while 53.16% of cases had no pathogen identified.

**Conclusions:**

Preliminary evidence suggests NI is associated with elevated psychiatric symptom burden at admission and significant symptom improvement during hospitalization, though residual symptoms persist. Without a non-infected control group, directionality cannot be established. Companion-accompanied patients represent a high-risk subgroup with elevated psychiatric symptom burden and distinct infection patterns. NI duration was independently associated with increased hospitalization costs. These findings highlight the need for integrated infection-psychiatric management protocols in psychiatric hospitals.

## 1 Introduction

Nosocomial infections (NIs), also termed healthcare-associated infections, remain a global public health concern, affecting approximately 7% of hospitalized patients in developed countries and up to 10% in developing regions (1, 2). Psychiatric hospitals present a unique ecological niche for NI due to their distinctive operational characteristics: closed ward environments, prolonged hospitalization, communal living arrangements, impaired patient self-care capacity, and limited infection control infrastructure (3, 4). Reported NI incidence rates in psychiatric settings range from 1.78% to 8.57%, with respiratory tract infections consistently dominating the infection profile, accounting for 53%–79% of all NIs (5–7).

The relationship between psychiatric disorders and infection susceptibility is increasingly recognized as bidirectional. On one hand, depression and anxiety may compromise immune function through dysregulated inflammatory pathways—elevated pro-inflammatory cytokines (IL-6, TNF-alpha, CRP) have been consistently documented in major depressive disorder (8, 9), potentially increasing infection susceptibility. On the other hand, acute infection as a psychological stressor may be associated with elevated psychiatric symptom burden through both direct neuroinflammatory mechanisms and indirect psychosocial pathways (10), though direct evidence of NI-driven symptom worsening from psychiatric settings remains limited. However, this bidirectional hypothesis has primarily been tested in general hospital settings, and evidence from psychiatric hospitals using validated psychiatric rating scales remains scarce.

Another underexplored dimension is the role of companion caregivers (family members or hired attendants who stay with patients throughout hospitalization), a practice common in Chinese psychiatric hospitals. Companion caregivers may serve as both protective factors (assisting with hygiene and feeding) and potential vectors for pathogen introduction from the community (5, 11). Furthermore, the economic burden imposed by NI in psychiatric settings—where prolonged hospitalization is already costly—has received insufficient attention (12).

This retrospective study of 79 NI cases from a psychiatric hospital in China aimed to: (1) characterize NI patterns and pathogen distribution; (2) characterize the trajectory of depressive and anxiety symptoms during hospitalization in NI patients using paired HAMD/HAMA analysis; (3) identify demographic risk factors for non-respiratory infections; (4) assess the impact of companion caregivers on both psychiatric symptoms and infection patterns; and (5) model the economic burden attributable to NI duration.

## 2 Materials and methods

### 2.1 Study design and setting

This retrospective study was conducted at the Third People’s Hospital of Foshan. The hospital provides inpatient psychiatric services including closed and open wards for patients with mood disorders, schizophrenia, and other mental illnesses. The study period spanned from March 2022 to June 2025. The study was approved by the Ethics Committee of the Third People’s Hospital of Foshan (Approval No: FSSY-LS202513). All data were anonymized, and patient confidentiality was strictly maintained. The research was conducted in accordance with the Declaration of Helsinki. Given the retrospective nature of this study and the use of fully anonymized electronic medical records, the requirement for informed consent was waived by the Ethics Committee. This study was reported following the Strengthening the Reporting of Observational Studies in Epidemiology (STROBE) guideline (13).

### 2.2 Study population

Seventy-nine patients who developed NI during their psychiatric hospitalization were included. NI was diagnosed according to the Chinese Ministry of Health’s “Diagnostic Criteria for Nosocomial Infection (Trial)”, which aligns with WHO definitions (14). Per these pre-specified criteria, a NI was confirmed when a patient developed a new infection after at least 48 h of hospitalization that was neither present nor incubating at admission. Diagnoses were established by infection control physicians on the basis of clinical signs and symptoms (fever, productive cough, dysuria), laboratory findings (leukocytosis, elevated C-reactive protein, positive culture or pathogen nucleic acid test results), and imaging evidence (chest radiography or computed tomography for respiratory infections) documented in the electronic medical records; all suspected cases were reviewed and confirmed by the Department of Infection Control. Because NI diagnosis relies on standardized clinical criteria rather than a single laboratory test, sensitivity and specificity cannot be directly computed in this retrospective setting; this is inherent to surveillance definitions and was addressed by strict adherence to the nationally mandated diagnostic standard and confirmation by trained infection control physicians. Inclusion criteria: (1) confirmed NI diagnosis; (2) infection acquired after at least 48 hours of hospitalization; (3) complete clinical records. Exclusion criteria: (1) infections present or incubating at admission; (2) critical data missing.

### 2.3 Data collection

The following data were extracted from electronic medical records: Demographics (age, sex, educational attainment, health insurance type, companion caregiver status); Infection characteristics (NI type, infection site, pathogen identification, NI duration, infection date); the interval from psychiatric hospital admission to NI onset (admission-to-infection interval) was calculated for each patient; Hospitalization data (length of stay, total hospitalization cost, patient self-pay amount, admission/discharge dates); Psychiatric symptom assessment (HAMD scores at admission and before discharge; HAMA total scores at admission and before discharge); Treatment data (antibiotic use, antibiotic regimen). Seasons were defined as: spring (March–May), summer (June–August), autumn (September–November), winter (December–February). The HAMD and HAMA are clinician-administered rating scales completed by trained psychiatrists. The HAMD (15) assesses the severity of depressive symptoms across domains including depressed mood, guilt, suicidal ideation, insomnia, psychomotor retardation or agitation, anxiety, and somatic symptoms; each item is scored on a 0–4 or 0–2 scale, yielding a total score ranging from 0 to 52, with higher scores indicating more severe depression. The HAMA (16) evaluates both psychic anxiety (anxious mood, tension, fears) and somatic anxiety (muscular, cardiovascular, respiratory, and gastrointestinal symptoms); each item is scored 0-4, yielding a total score ranging from 0 to 56, with higher scores indicating more severe anxiety. The Chinese version of the HAMD has demonstrated good reliability and validity (17), and both instruments are widely used in Chinese psychiatric settings.

### 2.4 Statistical analysis

All analyses were conducted using SPSS 26.0. Continuous variables were expressed as mean ± standard deviation or median with interquartile range; categorical variables as frequencies and percentages. Group comparisons employed independent samples t-test (or Mann-Whitney U test for non-normal distributions) for continuous variables and chi-square test for categorical variables. Paired comparisons of HAMD/HAMA scores used both paired t-test and Wilcoxon signed-rank test to account for potential non-normality. Effect sizes were calculated using Cohen’s d. Linear correlations were assessed using Pearson’s r. Multiple linear regression was performed to model total hospitalization cost as a function of NI duration, LOS, age, sex, companion status, infection type, and pathogen detection status. Additional regression analyses were performed for self-pay amount using the same predictor set. Pearson correlation was used to assess the relationship between admission-to-infection interval and total hospitalization cost. Seasonal distribution was analyzed using chi-square tests for season versus infection type. For multiple linear regression models, multicollinearity was assessed using variance inflation factors (VIF), with VIF < 5 considered acceptable. Model assumptions including normality of residuals and homoscedasticity were evaluated through residual analysis. Given the exploratory nature of this study, multiple comparisons were not formally corrected; however, the interpretive focus was on effect sizes and confidence patterns rather than isolated P-values. Effect sizes (Cohen’s d) were reported to facilitate interpretation of clinical significance. Missing data: paired HAMD and HAMA analyses were based on complete cases because discharge assessments were not available for all patients (owing to early discharge, transfer, or incomplete records); no other variables had missing values, and all regression models therefore included the full cohort. To assess potential selection bias, baseline characteristics of the paired subgroup were compared with those of the remaining cohort. Confounding control: both unadjusted (simple linear regression) and adjusted (multiple linear regression) estimates were computed and are reported with their 95% confidence intervals (95% CI); adjusted models included NI duration, LOS, age, sex, companion status, infection type, and pathogen detection status as covariates.

## 3 Results

### 3.1 Demographic characteristics

As shown in **Table 1**, among 79 NI cases, females predominated (44/79, 55.70%). Mean age was 41.86 ± 21.87 years (median 39.0). The age distribution was: ≤30 years (33, 41.77%), 31–60 years (26, 32.91%), >60 years (20, 25.32%). Educational attainment was predominantly junior high school (35, 44.30%). Health insurance was primarily resident medical insurance (48, 60.76%). Half of the patients had companion caregivers (40/79, 50.63%).

**Table 1:** Demographic characteristics of 79 NI patients.

Variable	Category	n (%)
Sex	Female	44 (55.70)
	Male	35 (44.30)
Age group	no more than 30 years	33 (41.77)
	31-60 years	26 (32.91)
	>60 years	20 (25.32)
Education	Junior high school	35 (44.30)
	Primary school	17 (21.52)
	Senior high school	13 (16.46)
	Others	14 (17.72)
Insurance	Resident medical insurance	48 (60.76)
	Employee medical insurance	24 (30.38)
	Uninsured/others	7 (8.86)
Companion caregiver	Yes	40 (50.63)
	No	39 (49.37)

Data are presented as n (%). ± denotes standard deviation (SD).

### 3.2 Infection type and site distribution

As shown in **Table 2**, respiratory tract infections were the dominant NI type (60/79, 75.95%), followed by urinary tract infections (11/79, 13.92%), other site infections (4/79, 5.06%), mixed respiratory-urinary infections (2/79, 2.53%), and gastrointestinal infections (2/79, 2.53%). Among respiratory infections, bilateral lung involvement was most frequent (27/60, 45.00%), followed by right lung (20/60, 33.33%), upper respiratory tract (8/60, 13.33%), and left lung (5/60, 8.33%).

**Table 2:** Distribution of NI types and sites.

NI type	n (%)	Primary sites
Respiratory tract infection	60 (75.95)	Bilateral lung (27), right lung (20), upper respiratory (8), left lung (5)
Urinary tract infection	11 (13.92)	Urinary tract (11)
Other site infection	4 (5.06)	Other sites (2), skin (1), periodontal abscess (1)
Mixed respiratory-urinary	2 (2.53)	Bilateral lung+urinary (1), upper respiratory+urinary (1)
Gastrointestinal infection	2 (2.53)	Gastrointestinal (2)

### 3.3 Pathogen identification

No pathogen was identified in 42/79 cases (53.16%). Among identified pathogens, Mycoplasma pneumoniae was most frequent (14, 17.72%), followed by Klebsiella spp. (6, 7.59%), Escherichia coli (5, 6.33%), Staphylococcus spp. (4, 5.06%), SARS-CoV-2 (3, 3.80%), influenza virus (2, 2.53%), and others (3, 3.80%). Respiratory infections yielded Mycoplasma pneumoniae as the primary identified pathogen, while urinary tract infections were predominantly caused by E. coli (5/11, 45.45%).

### 3.4 Demographic risk factors for non-respiratory infections

When NI was dichotomized into respiratory vs. non-respiratory, two demographic factors showed significant associations (**Table 3**): (1) Sex—females had significantly higher non-respiratory infection rates (16/44, 36.36% vs 3/35, 8.57%, chi-square=6.792, P = 0.009); (2) Companion caregiver status—accompanied patients had higher non-respiratory infection rates (14/40, 35.00% vs 5/39, 12.82%, chi-square=4.173, P = 0.041). Age group (chi-square=5.474, P = 0.065), educational attainment (P = 0.568), and insurance type (P = 0.051) showed no significant association.

**Table 3:** Demographic factors associated with NI type.

Factor	Chi-square	P-value
Sex	6.792	0.009*
Age group	5.474	0.065
Companion caregiver	4.173	0.041*
Education	4.811	0.568
Insurance type	5.973	0.051

NI was dichotomized into respiratory vs. non-respiratory (respiratory: respiratory tract infection only; non-respiratory: urinary tract, other site, gastrointestinal, and mixed respiratory-urinary infections). Educational attainment was analyzed across seven categories (illiterate, primary school, junior high school, senior high school, technical secondary school, junior college, bachelor's degree or above). Chi-square test was used for categorical variable comparisons. *P < 0.05 was considered statistically significant.

### 3.5 Paired HAMD/HAMA analysis: symptom trajectory during hospitalization

Among patients with paired admission and discharge HAMD scores (the remaining patients lacked discharge assessments due to early discharge, transfer, or incomplete records), admission scores were 23.53 ± 10.13 and discharge scores were 16.53 ± 10.94, yielding a significant improvement of 7.00 ± 10.60 points (paired t=2.723, P = 0.015; Wilcoxon signed-rank test P = 0.015). Paired analysis of patients with both admission and discharge HAMA scores also revealed a significant decrease: admission 15.69 ± 6.64 vs. discharge 11.50 ± 7.32, mean change 4.19 ± 6.32, paired t=2.652, P = 0.018; Wilcoxon signed-rank test P = 0.029 (**Table 4**).

**Table 4:** Paired comparison of HAMD and HAMA scores.

Scale	Admission	Discharge	P	Cohen d
HAMD	23.53±10.13	16.53±10.94	0.015*	0.66
HAMA	15.69±6.64	11.50±7.32	0.018*	0.66

Paired t-test and Wilcoxon signed-rank test were used for within-subject comparisons. HAMD, Hamilton Depression Rating Scale (17-item); HAMA, Hamilton Anxiety Rating Scale. *P<0.05 was considered statistically significant.

These results demonstrate that NI patients showed significant symptom improvement from admission to discharge with medium effect sizes, though residual moderate symptom burden persisted at discharge, suggesting incomplete recovery. Without a non-infected control group, the directionality of the NI-symptom relationship cannot be definitively established. HAMD/HAMA change scores showed no significant correlation with NI duration (HAMD: r=0.248, P = 0.338; HAMA: r=0.098, P = 0.718), suggesting that the elevated admission symptom burden reflects pre-existing severity rather than NI-driven accumulation, and that symptom improvement during hospitalization is not contingent on NI duration.

### 3.6 Companion caregiver impact on psychiatric symptoms

Companion-accompanied patients exhibited significantly higher HAMD admission scores than non-accompanied patients (25.47 ± 6.98 vs. 18.22 ± 11.02, t=2.486, P = 0.017). Similarly, HAMA admission scores were significantly higher in the companion group (17.50 ± 5.98 vs. 11.83 ± 7.52, t=2.617, P = 0.013). Furthermore, companion-accompanied patients had a significantly shorter admission-to-infection interval (median 13.0 vs. 28.0 days, Mann-Whitney U test P = 0.003); one patient with a negative interval resulting from an inconsistent infection date was excluded from this analysis.

### 3.7 Companion vs. non-companion group comparisons

As detailed in **Table 5**, companion-accompanied patients were significantly older (50.58 ± 25.19 vs. 32.92 ± 12.97 years, P = 0.0002), had shorter LOS (39.19 ± 31.18 vs. 71.37 ± 44.95 days, P = 0.0004), tended to have lower total hospitalization costs (22946.86 ± 14508.75 vs. 29513.64 ± 15229.01 CNY, P = 0.053), and had significantly lower self-pay amounts (6190.64 ± 3665.18 vs. 9315.00 ± 6198.32 CNY, P = 0.008). NI duration was comparable between groups (9.47 ± 6.02 vs. 9.15 ± 5.55 days, P = 0.806).

**Table 5:** Clinical parameters by companion caregiver status.

Parameter	Companion(n=40)	Non-companion(n=39)	P
Age (years)	50.58±25.19	32.92±12.97	0.0002*
LOS (days)	39.19±31.18	71.37±44.95	0.0004*
Total cost (CNY)	22946.86±14508.75	29513.64±15229.01	0.053
Self-pay (CNY)	6190.64±3665.18	9315.00±6198.32	0.008*
NI duration(days)	9.47±6.02	9.15±5.55	0.806
Admission-to-infection(days)	13.0 (8.0-21.8)	28.0 (15.0-56.5)	0.003*

LOS, length of stay; NI, nosocomial infection; CNY, Chinese Yuan. ± denotes standard deviation. Independent-samples t-test was used for continuous variable comparisons; the admission-to-infection interval is reported as median (interquartile range) and was compared using the Mann-Whitney U test; one patient with a negative interval resulting from an inconsistent infection date was excluded from this analysis. *P < 0.05 was considered statistically significant.

### 3.8 Seasonal distribution and antibiotic usage

As shown in **Table 6**, spring had the highest NI case volume (31/79, 39.24%), followed by winter (18, 22.78%), autumn (15, 18.99%), and summer (15, 18.99%). April was the peak month (17 cases). No significant association was found between season and infection type (chi-square=1.338, P = 0.720). Antibiotics were used in 75/79 cases (94.94%). The most common regimens were ceftriaxone 2g (12 cases), levofloxacin 0.5g oral (11 cases), and cefuroxime axetil 0.5g (6 cases).

**Table 6:** Seasonal distribution and antibiotic usage of NI cases.

Variable	Category	n (%)
Season	Spring	31 (39.24)
	Winter	18 (22.78)
	Autumn	15 (18.99)
	Summer	15 (18.99)
Peak month	April	17 (21.52)
Season × Infection type	Chi-square=1.338, P=0.720	
Antibiotic usage	Yes	75 (94.94)
	No	4 (5.06)
Top 3 regimens	Ceftriaxone 2g	12 (15.19)
	Levofloxacin 0.5g oral	11 (13.92)
	Cefuroxime axetil 0.5g	6 (7.59)

NI, nosocomial infection. Season was defined as: spring (March-May), summer (June-August), autumn (September-November), winter (December-February). No significant association was found between season and infection type.

### 3.9 Economic burden analysis

As detailed in **Table 7**, mean total hospitalization cost was 26,188.69 CNY (median 22,211.14), mean self-pay amount 7,733.05 CNY (median 6,220.90), mean LOS 55.08 days (median 38.46), and mean NI duration 9.32 days (median 7.0, range 2-26).

**Table 7a:** Descriptive statistics of economic burden and hospitalization outcomes.

Parameter	Mean ± SD	Median (IQR)	Range
Total hospitalization cost (CNY)	26,188.69 ± 15,137.93	22,211.14 (16,685.46–30,673.40)	5,725.07–81,027.66
Self-pay amount (CNY)	7,733.05 ± 5,282.53	6,220.90 (4,195.10–10,211.92)	6.06–29,990.68
LOS (days)	55.08 ± 41.63	38.46 (23.72–71.88)	5.00–182.74
NI duration (days)	9.32 ± 5.76	7.00 (5.50–12.50)	2.00–26.00

Data are presented as mean ± standard deviation (SD) and median (interquartile range, IQR). CNY = Chinese Yuan; LOS = length of stay; NI = nosocomial infection. N = 79.

**Table 7b:** Regression analysis of economic burden attributable to NI.

Variable	β	SE	95% CI	P	R²
Simple linear regression					0.077
NI duration → total cost (CNY/day)	730.79	287.74	(157.84, 1303.75)	0.013	
Multiple linear regression (total cost)					0.774
NI duration (days)	507.45	156.48	(195.44, 819.46)	0.002	
LOS (days)	307.99	23.43	(261.27, 354.72)	<0.001	
Age (years)	19.57	46.10	(−72.35, 111.48)	0.673	
Sex (female)	1,704.59	2,021.72	(−2,326.60, 5,735.77)	0.402	
Companion status (yes)	3,889.95	2,101.88	(−301.08, 8,080.98)	0.068	
Respiratory infection type	5,272.08	2,278.35	(729.17, 9,814.98)	0.024	
Pathogen detection (yes)	4,050.57	1,887.72	(286.56, 7,814.59)	0.035	
Multiple linear regression (self-pay)					0.234
NI duration (days)	100.00	93.56	(−86.38, 286.38)	0.289	
LOS (days)	33.87	12.92	(8.14, 59.61)	0.011	
Age (years)	−96.64	24.51	(−145.46, −47.81)	<0.001	
Pearson correlation					
Admission-to-infection interval vs. total cost	r = 0.405	—	—	<0.001	

β = unstandardized regression coefficient; SE = standard error; CI = confidence interval; R² = coefficient of determination. Simple linear regression: total cost = 730.79 × NI duration (days) + intercept. Multiple linear regression for total cost (n = 79): adjusted for NI duration, LOS, age, sex, companion status, infection type, and pathogen detection. Multiple linear regression for self-pay (n = 79): predictors included NI duration, LOS, and age. All variance inflation factors (VIF) were < 2.0, indicating no significant multicollinearity. Self-pay regression: older patients had lower self-pay amounts, consistent with higher insurance coverage rates in the elderly group.

Simple linear regression showed that each additional day of NI duration increased total hospitalization cost by 730.79 CNY (95% CI: 157.84-1303.75, R-squared=0.077, Pearson r=0.278, P = 0.013). Multiple linear regression for total cost prediction yielded R-squared=0.774, with the following adjusted coefficient estimates (95% CI): NI duration (beta=507.45, 95% CI 195.44-819.46, P = 0.002), LOS (beta=307.99, 95% CI 261.27-354.72, P<0.001), age (beta=19.57, 95% CI -72.35-111.48, P = 0.673), sex (beta=1704.59, 95% CI -2326.60-5735.77, P = 0.402), companion status (beta=3889.95, 95% CI -301.08-8080.98, P = 0.068), respiratory infection type (beta=5272.08, 95% CI 729.17-9814.98, P = 0.024), and pathogen detection (beta=4050.57, 95% CI 286.56-7814.59, P = 0.035).

Self-pay cost regression (R-squared=0.234) showed NI duration (beta=100.00, 95% CI -86.38-286.38, P = 0.289), LOS (beta=33.87, 95% CI 8.14-59.61, P = 0.011), and age (beta=-96.64, 95% CI -145.46 to -47.81, P<0.001) as predictors, with older patients having lower self-pay amounts. The admission-to-infection interval was positively correlated with total hospitalization cost (r=0.405, P<0.001).

## 4 Discussion

Overall, this study generated five principal findings corresponding to the pre-specified objectives. First, respiratory tract infections predominated, with Mycoplasma pneumoniae as the most frequently identified pathogen. Second, paired analyses revealed significant improvement in depressive and anxiety symptoms during hospitalization with medium effect sizes, yet with residual symptom burden at discharge. Third, female sex and companion caregiver status—but not age, education, or insurance type—were significantly associated with non-respiratory infection. Fourth, companion-accompanied patients exhibited elevated admission symptom burden and earlier infection onset. Fifth, NI duration independently predicted total hospitalization cost. With respect to the bidirectional hypothesis, our data support the susceptibility arm: the elevated admission symptom burden in NI patients, particularly among companion-accompanied patients, is consistent with increased susceptibility to infection in more severely symptomatic patients. However, the hypothesis that NI itself drives progressive symptom deterioration was not supported: symptoms improved significantly during hospitalization, and the magnitude of improvement was unrelated to NI duration.

Respiratory tract infections accounted for 75.95% of all NIs, consistent with previous reports from psychiatric hospitals in China (53%–79%) (5–7) and internationally (3, 5). This dominance likely reflects multiple converging factors: closed ward environments with limited ventilation facilitating aerosolized pathogen transmission; impaired cough reflex and swallowing dysfunction in psychiatric patients, particularly those on antipsychotic medications such as clozapine (18); communal dining and living arrangements increasing cross-infection opportunities; and seasonal respiratory pathogen activity amplified by closed ward ecology.

The paired HAMD/HAMA analysis reveals a nuanced picture: NI patients exhibited significant symptom improvement from admission to discharge, yet retained substantial residual symptom burden at discharge. While this improvement direction does not directly demonstrate NI-driven symptom worsening, the high admission symptom burden in NI patients — significantly elevated in the companion subgroup — suggests a severity-NI susceptibility association consistent with the depression-infection arm of the bidirectional hypothesis (19). The persistence of moderate residual symptoms despite treatment may reflect an NI-associated attenuation of recovery trajectory, though this requires confirmation with a non-infected control group. Taken together, our data are more consistent with a susceptibility model than with NI-driven symptom accumulation; whether NI attenuates the recovery trajectory—as suggested by the persistent residual symptoms—warrants controlled studies.

Several mechanisms may theoretically contribute to elevated symptom burden and incomplete recovery in NI patients. First, infection-induced systemic inflammation activates the kynurenine pathway, shifting tryptophan metabolism from serotonin synthesis toward quinolinic acid production, a neurotoxic NMDA receptor agonist that exacerbates depressive symptoms (20). Second, the psychological stress of being infected—fever, pain, isolation, and perceived health threat—directly activates stress-response pathways, increasing cortisol and sympathetic tone (19). Third, NI often necessitates antibiotic treatment, which may disrupt the gut microbiome and affect the microbiota-gut-brain axis (21). These mechanisms remain speculative in the present dataset and require prospective studies with non-infected controls for validation. Importantly, the magnitude of symptom improvement was not correlated with NI duration (HAMD: r=0.248, P = 0.338; HAMA: r=0.098, P = 0.718), suggesting that the admission symptom burden reflects pre-existing severity rather than NI-driven accumulation. This implies that early infection detection and prompt treatment may mitigate psychiatric symptom deterioration.

Companion caregivers represent a distinctive feature of Chinese psychiatric hospitalization. Our data reveal a complex dual role: As a risk marker, companion-accompanied patients had significantly higher HAMD and HAMA admission scores, were older, and had higher non-respiratory infection rates. This suggests that companion assignment is clinically driven—more impaired, elderly patients require caregiver support. As potential vectors, companion-accompanied patients developed NI significantly earlier. Caregivers traversing between community and hospital may introduce pathogens (5, 22). However, companions also facilitated shorter LOS and lower self-pay costs, suggesting benefits in care coordination. These findings argue for structured companion caregiver infection control training.

Females had significantly higher non-respiratory infection rates, driven primarily by urinary tract infections. This anatomical predisposition is well-established (23). Female psychiatric inpatients face compounded urinary infection risk: anatomical susceptibility, psychotropic medication effects on bladder function, and impaired hygiene. In contrast, age was not significantly associated with non-respiratory infection risk in this cohort (chi-square=5.474, P = 0.065), despite the well-recognized effects of immunosenescence and multi-morbidity on infection susceptibility in elderly patients (24); the modest number of elderly patients in this sample may have limited statistical power to detect such an association.

Mycoplasma pneumoniae dominance reflects recent epidemic trends in China (25). The 53.16% unidentified pathogen rate points to inadequate sampling in psychiatric settings (26). The detection of SARS-CoV-2 and influenza virus underscores closed ward vulnerability to epidemic respiratory pathogens (27, 28). The linear relationship between NI duration and total cost provides a quantifiable metric for cost-effectiveness analysis. The multiple regression model demonstrates that NI duration, LOS, infection type, and pathogen detection collectively explain 77% of cost variance, consistent with established LOS-infection cost literature (29, 30). The negative age coefficient in self-pay regression reflects higher insurance coverage rates among elderly patients, resulting in lower out-of-pocket proportions despite greater total costs.

The near-universal antibiotic use rate (94.94%) in this cohort raises important antimicrobial stewardship considerations. While empirical antibiotic initiation may be justified in acutely ill psychiatric patients with suspected bacterial infection, the high prescription rate combined with a 53.16% pathogen-negative rate suggests potential for antimicrobial overuse. This is particularly concerning given the global threat of antimicrobial resistance and the limited diagnostic capacity in psychiatric settings. The predominance of broad-spectrum agents (ceftriaxone, levofloxacin) without confirmed pathogen identification highlights the need for antimicrobial stewardship programs tailored to psychiatric hospitals, including standardized empirical therapy protocols, de-escalation pathways guided by microbiological results, and regular audit and feedback mechanisms. Furthermore, the detection of SARS-CoV-2 and influenza virus in this cohort underscores the importance of maintaining respiratory virus surveillance and outbreak preparedness protocols in closed psychiatric wards, which remain highly vulnerable to epidemic respiratory pathogen transmission.

From an infection prevention and control perspective, our findings highlight several actionable priorities for psychiatric hospitals. First, the predominance of respiratory infections (75.95%) warrants enhanced ventilation, air disinfection, and respiratory hygiene protocols in closed wards. Second, the significantly higher non-respiratory infection rates among female patients call for targeted surveillance programs, including regular urinary tract infection screening for at-risk subgroups and assessment of catheter utilization where applicable. Third, the earlier infection onset in companion-accompanied patients suggests that structured infection control training and health screening for caregivers entering the ward should be implemented. Fourth, the low pathogen detection rate (46.84%) indicates an urgent need to improve microbiological sampling protocols and laboratory capacity in psychiatric settings, which is essential for both targeted therapy and resistance surveillance.

### Limitations

Several limitations merit acknowledgment, along with the measures taken to mitigate their impact. (1) The small sample size (n=79) with all cases being NI-positive precluded incidence rate calculation and logistic regression for NI risk factors; limited paired HAMD/HAMA data reduced statistical power; multiple statistical comparisons were performed without formal correction; and the single-center retrospective design limits generalizability. To mitigate this, effect sizes and 95% confidence intervals were reported alongside P-values, both parametric (paired t-test) and non-parametric (Wilcoxon signed-rank test) approaches were applied and yielded concordant results, key confounders were adjusted in multivariable regression, the study setting and procedures were described in detail to facilitate comparability with other centers, and key findings were consistent with previous multi-center and international reports. (2) The absence of a non-infected control group precluded direct psychiatric symptom comparison and establishment of causality. To mitigate this, we used strictly descriptive language for symptom change, avoided causal claims, and explicitly acknowledged that directionality cannot be established from this design. (3) Data quality was limited by the low pathogen detection rate (53.16% unidentified) and the use of the 2001 Chinese diagnostic criteria for nosocomial infection, which may differ from current international definitions and have been noted to require updating (14). To mitigate this, our detection rate was benchmarked against prior psychiatric hospital studies with enhanced microbiological sampling identified as an actionable priority, and the applied criteria are the nationally mandated standard aligning with WHO definitions. (4) The paired HAMD/HAMA subgroup may not be representative of the full cohort, and data on antimicrobial resistance patterns, device-associated infections, and antibiotic prescribing appropriateness were not available. To mitigate this, we compared the paired subgroup with the remaining cohort: although the paired subgroup was significantly younger (28.18 ± 15.20 vs. 45.61 ± 22.01 years, P = 0.003), admission HAMD and HAMA scores were comparable (P = 0.283 and P = 0.348, respectively), and symptom improvement was not associated with age (P = 0.479); the age imbalance nonetheless warrants caution when generalizing the paired findings, and the identified data gaps are prioritized for future prospective surveillance.

## 5 Conclusions

This retrospective study provides preliminary evidence that psychiatric inpatients with nosocomial infection carry elevated depressive and anxiety symptom burden at admission and show significant improvement during hospitalization with medium effect sizes, though residual symptoms persist at discharge. Without a non-infected control group, the directionality of the NI-symptom relationship cannot be definitively established. Companion caregivers emerge as a clinically significant factor associated with both elevated psychiatric symptom burden and earlier infection onset. Respiratory tract infections remain dominant, but female sex and companion status significantly increase non-respiratory infection risk. Each additional day of NI duration adds approximately 731 CNY to total hospitalization cost. These findings advocate for: integrated infection-psychiatric management protocols with symptom trajectory monitoring; structured infection control training for companion caregivers; enhanced pathogen identification and antimicrobial stewardship; and early NI detection to minimize both psychiatric symptom burden and economic burden. Prospective controlled studies are needed to confirm these preliminary findings.

## Data Availability

The original contributions presented in the study are included in the article/supplementary material. Further inquiries can be directed to the corresponding authors.
